# European beech reproduction is not reduced by drought, including the 2003, 2018, and 2022 extremes

**DOI:** 10.1073/pnas.2607167123

**Published:** 2026-06-29

**Authors:** Jakub Szymkowiak, Michał Bogdziewicz, Dave Kelly, Jessie Foest, Sabine Braun, Burkhard Beudert, Francesco Chianucci, Andrea Cutini, Rachel Gaulton, Georg Gratzer, Angelika Kölbl, Georges Kunstler, Jonathan G. A. Lageard, Henning Meesenburg, Francesco Mezzavilla, Martina Mund, Anita Nussbaumer, Mario B. Pesendorfer, Wolfgang Schmidt, Anne Thimonier, Peter A. Thomas, Stanislav Vacek, Zdeněk Vacek, Arne Verstraeten, Markus Wagner, Andrew Hacket-Pain

**Affiliations:** ^a^Forest Biology Center, Institute of Environmental Biology, Faculty of Biology, Adam Mickiewicz University, Poznań 61-614, Poland; ^b^Population Ecology Research Unit, Institute of Environmental Biology, Faculty of Biology, Adam Mickiewicz University, Poznań 61-614, Poland; ^c^ School of Biological Sciences, University of Canterbury, Christchurch 8140, New Zealand; ^d^Institute for Applied Plant Biology, Witterswil, Switzerland; ^e^ Department of Conservation and Research, Bavarian Forest National Park, Grafenau, Germany; ^f^ Consiglio per la ricerca in agricoltura e l’analisi dell’economia agraria (CREA), Centro di Ricerca Foreste e Legno, Arezzo 52100, Italy; ^g^Fera Science Ltd., York Biotech Campus, Sand Hutton, York YO41 1LZ, United Kingdom; ^h^ Department of Ecosystem Management, Climate and Biodiversity, Institute of Forest Ecology, BOKU University, Vienna A-1190, Austria; ^i^ Université Grenoble Alpes, Institut national de recherche pour l’agriculture, l’alimentation et l’environnement (INRAE), Laboratoire EcoSystémes et Sociétés En Montagne (LESSEM), St-Martin-d’Héres F-38402, France; ^j^ Department of Natural Sciences, Manchester Metropolitan University, Manchester M1 5GD, United Kingdom; ^k^ Northwest German Forest Research Institute, Department of Environmental Control, Section Intensive Environmental Monitoring, Göttingen 37079, Germany; ^l^Via Malviste 4, Silea (TV) 31057, Italy; ^m^Forestry Research and Competence Centre Gotha, Gotha, Germany; ^n^ Swiss Federal Institute for Forest, Snow and Landscape Research WSL, Land Change Science, Birmensdorf CH-8903, Switzerland; ^o^ Department of Silviculture and Forest Ecology of the Temperate Zones, University of Göttingen, Büsgenweg 1, Göttingen D-37077, Germany; ^p^ School of Life Sciences, Keele University, Staffordshire ST5 5BG, United Kingdom; ^q^ Department of Silviculture, Faculty of Forestry and Wood Sciences, Czech University of Life Sciences Prague, Prague, Praha - Suchdol 165 00, Czech Republic; ^r^ Research Institute for Nature and Forest, Environment and Climate Unit, Geraardsbergen BE-9500, Belgium; ^s^ Department of Geography and Planning, School of Environmental Sciences, University of Liverpool, Liverpool L69 7ZT, United Kingdom

**Keywords:** climate change, drought, seed production, tree demography, forest resilience

## Abstract

Climate change is intensifying drought stress in temperate forests, but its effects on tree reproduction, central to forest regeneration and migration capability, remain poorly understood. Here, we analyze 221 time series of beech (*Fagus sylvatica*) seed production across Europe to test whether drought reduces seed output. We isolate drought exposure during the flowering, pollination, and seed maturation phases of reproduction, and test for legacy effects on future reproduction. Seed production was not impaired by summer drought, and dry spring conditions were associated with increased output, likely via enhanced pollen dispersal. Thus, once initiated, beech reproduction is not reduced by drought, with no suppression of reproduction the following year. Reproduction was not reduced at the driest sites during exceptional European summer droughts in 2003, 2018, and 2022. Considered alongside prior evidence that drought suppresses forest growth and elevates mortality, these findings indicate that vital rates can respond in opposite directions to the same stressor. Such contrasts may sustain forest reproduction during heat–drought events yet shift demographic balance toward higher mortality and turnover as climatic extremes intensify.

## Significance

Forest resilience to climate change depends not only on whether trees survive droughts, but also on whether they can still reproduce. Long-term seed production records from across Europe reveal that European beech continues to produce seeds even during the most severe recent summer droughts, including the 2003, 2018, and 2022 extremes. This resilience of reproduction sharply contrasts with the drought-induced declines in growth and increases in mortality observed in the same species and regions. Our results demonstrate that tree demographic rates respond differently to drought, with implications for predicting forest regeneration, turnover, and long-term responses to a warmer, drier climate.

Climate change is altering average climatic conditions and their variability, leading to more frequent and intense extremes such as heatwaves and severe droughts (1). Observations across continents reveal consistent trends with increasing rates of forest disturbance, elevated tree mortality, and changing growth dynamics in adult trees and seedlings (2–6). In Europe, recent summer droughts have reached intensities without precedent in the past two millennia (7, 8), exerting mounting pressure on forest demography (3, 9). For instance, tree canopy mortality trends have accelerated in response to prolonged drying (10). The 2018–2020 European drought sharply illustrates the magnitude of current climatic pressures: stem growth declined by approximately 40% at sites in German forests (11, 12), and widespread dieback and mortality occurred among adult trees and saplings (13–15). Yet while there are published data on the demographic consequences of drought through growth and mortality, much less is known about how drought influences reproduction, despite its key importance for long-term forest dynamics (2, 16).

The resilience of forests to disturbance, including the potential reorganization to nonforest, depends on the magnitude and consistency of tree reproduction and subsequent regeneration (2, 16–18). In forests, tree reproduction is realized through seed production, a multistage process from floral initiation through pollination to seed maturation, which ultimately determines the potential for regeneration (19–21). In contrast to mortality, where extensive evidence and mechanistic theory link drought to hydraulic failure, carbon starvation, and biotic attack (22–25), reports of reproductive responses are sparse (26–30). This uncertainty is consequential: The direction and magnitude of reproductive responses will determine the regeneration potential of forests under drought (31), the capability of trees to track moving climate envelopes (32, 33), and, through the costs of reproduction, changes in the mortality risk to adult trees (34).

Drought is expected to influence tree seed production through multiple pathways. Drought can directly constrain photosynthesis and water uptake (35, 36), reducing the resources available for reproductive investment, which includes structural tissues and the carbon and nutrient-rich endosperm and embryo. Such resource limitation has been linked to declines in seed production in Scots pine (37), and is well documented in herbaceous systems (38). However, the consequences for seed output depend not only on resource availability, but also on how trees allocate those resources under stress. Reproduction can be prioritized or deprioritized under drought, relative to growth and other functions, including defense (34, 39, 40). For example, Lauder (34) suggested that trees may respond to drought by either allocating resources toward survival-related functions such as growth and defense, or by maintaining investment in reproduction at the potential cost of reduced survival. In support, in *Pinus ponderosa*, *Picea abies*, and *Quercus ilex*, drought makes the trade-off between reproduction and growth or defense more pronounced (40–42). Flowers are sensitive to drought-induced embolism (30), and drought can reduce pollen viability or fertilization rates (43), and trigger abortion of maturing fruits (27, 44). However, not all effects are negative: Warm and dry spring conditions may improve pollen dispersal and enhance pollination efficiency (45), potentially increasing seed set under some circumstances. Moreover, maintaining reproduction under drought may confer selective advantages if stress signals favorable postdisturbance conditions for recruitment or if completing reproduction carries lower short-term costs than reallocating resources (34, 46). Because these mechanisms can act at different stages of reproduction and can operate in opposite directions, inference from short records or single drought events is limited. Long-term seed production datasets spanning multiple drought and nondrought years are needed to separate drought effects occurring during flowering and pollination from those during seed maturation, and to test for lagged effects on subsequent reproduction.

Here, we analyze 221 time series (5,362 population-level annual observations) of European beech reproduction to test whether drought disrupts seed production. Reproduction in beech varies strongly among years (masting), largely as a result of temperature cues which regulate annual flowering effort (47, 48). Thus, we ask whether drought (climatic water balance, CWB) reduces seed output in years when trees are already committed to reproduce. By explicitly modeling the temperature cues that govern annual flowering effort, we separate cue-driven commitment from drought acting as a climatic veto during subsequent reproductive processes. We partitioned drought exposure into two phenological phases: flowering and pollination (spring, April–May) and fruit maturation (summer, June–September), and also tested for lagged effects of summer drought on seed production the following year. We hypothesized that spring drought could enhance seed production by promoting pollen dispersal under dry conditions in this wind-pollinated species (45, 49). In contrast, summer drought was expected to reduce seed production if trees allocate limited resources to competing sinks such as stem or root growth or storage under stress (34, 50). Alternatively, reproductive output could be maintained despite summer drought if finalizing seed maturation under stress is selectively advantageous, or a species lacks a regulatory mechanism to abort fruits (51). Two mechanisms could support such selective benefits. First, a form of reproductive persistence, akin to a “flight” strategy, where trees maintain reproduction at the expense of growth and survival-related functions (34). Second, reproduction may be favored under stress as an adaptive response to environmental signals (52, 53): Drought-induced canopy opening may signal favorable conditions for seedling establishment (46, 54), thus favoring investment in current seed maturation. In addition to analyzing seed production responses across the full range of drought conditions represented in our dataset, we also test whether recent exceptional spring/summer drought events in Europe (in 2003, 2018, and 2022) (55) were associated with declines in seed production, paralleling established responses of tree growth and survival to these events.

## Results

**Fig. 1.**
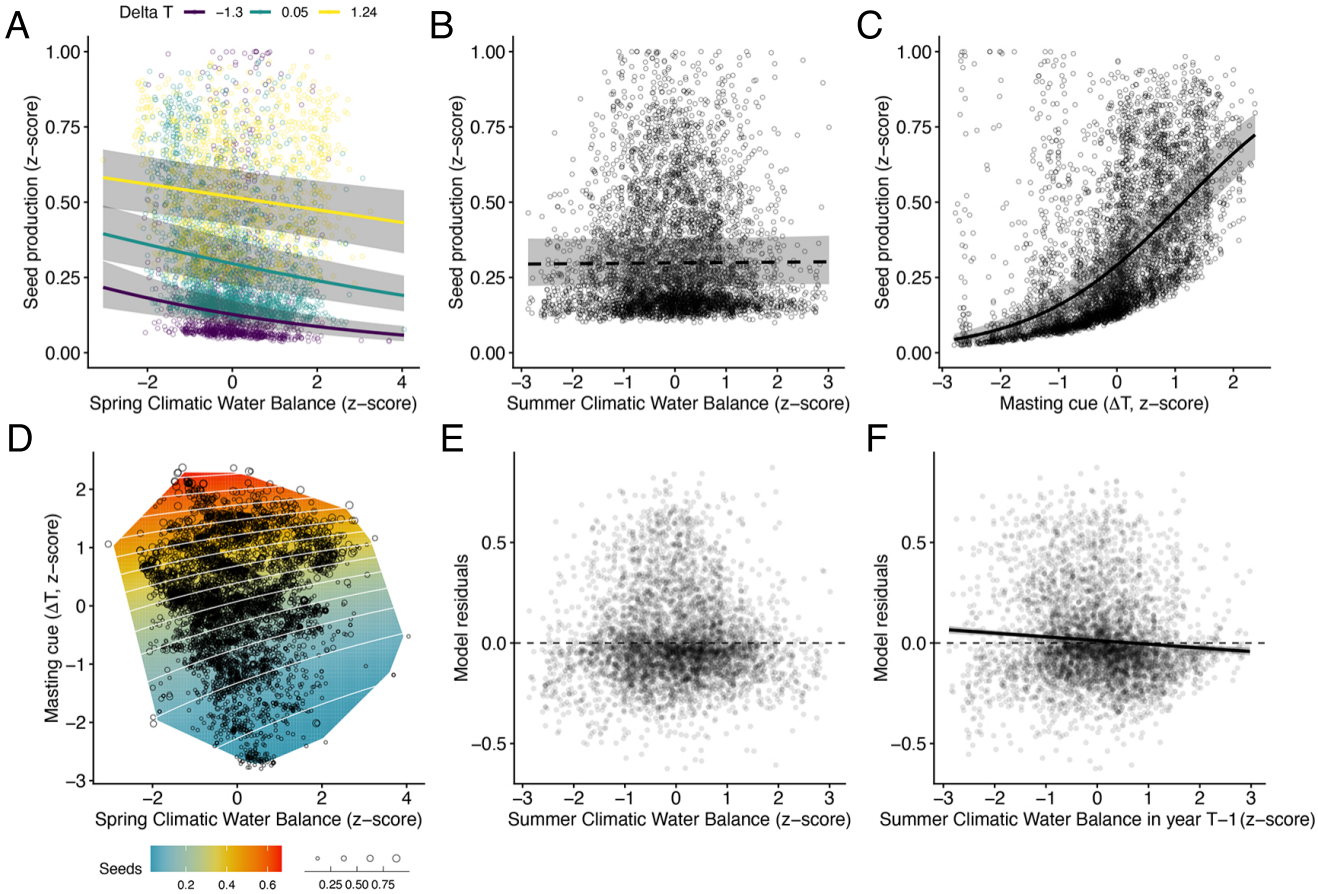
European beech reproduction is not suppressed by seasonal drought. Estimated relationships between (*A*) spring (April–May) climatic water balance (CWB, negative values indicate water deficit), (*B*) summer (July–September) CWB, and (*C*) masting cue (ΔT, i.e., difference between summer temperatures two and one year before flowering, see *Materials and Methods*) on population-level seed production in European beech. Colors in (*A*) indicate the conditional relationships for selected levels of ΔT, and the dashed line at (*B*) highlights a nonsignificant effect. Points on (*A*–*C*) show partial residuals of a model including the ΔT masting cue, spring CWB, summer CWB and prior year summer CWB and seed production (*Materials and Methods*). (*D*) Surface plot shows estimated population-level seed production effort across combinations of masting cue and spring CWB, with the convex hulls (parameter space across which predictions are computed) defined by observations (black circles). Points show population-level annual seed production. (*E*) Model residuals plotted against summer CWB highlight the lack of overlooked effects at extremes. (*F*) Model residuals plotted against summer CWB in year T–1 show the weak negative effect of past-year drought on seed production. Estimates are derived from a GLMM that included site as a random intercept (*N* sites = 221, *N* observations = 5,362).

European beech reproduction was not suppressed by seasonal drought during reproduction, with no evidence that spring or summer climatic water deficits suppressed seed production (Fig. 1, Table 1; results were consistent when using VPD to quantify drought, see *Materials and Methods* and *SI Appendix*, Table S1 and Fig. S1) Once trees were committed to flowering, as indicated by strong responses to the flowering cue (ΔT, i.e., the difference between June–July temperatures one and two years before seedfall; see *Materials and Methods*) (Fig. 1), seed production increased under spring drought (Fig. 1). The positive effect of spring drought was slightly more pronounced when the ΔT cue was weaker (Spring CWB and ΔT interaction, Table 1, Fig. 1). Seed production was insensitive to summer drought conditions irrespective of the ΔT cue (Fig. 1). The positive effect of ΔT on seed production was 5 times stronger than the effect of spring drought (climatic water balance, CWB), underscoring that reproductive output in beech is primarily governed by sensitivity to its masting cue. Residual analyses showed no systematic patterns with summer drought intensity, indicating that no additional effects were overlooked at the extremes (Fig. 1*F*). Summer CWB did not suppress reproduction the following year; a weak significant effect indicated seed production was in fact slightly higher in the year following summer drought.

**Table 1.** Results of the Generalized Linear Mixed model testing for the effects of seasonal drought on European beech reproduction

Term	Estimate	SE	z	*P*
Intercept	−1.37	0.039	−35.51	**<0.001**
ΔT	0.72	0.024	30.48	**<0.001**
Spring CWB	−0.16	0.018	−8.60	**<0.001**
Summer CWB	0.01	0.020	0.47	0.637
Summer CWB T–1	−0.17	0.022	−7.70	**<0.001**
Seeds T–1	−0.58	0.021	−27.73	**<0.001**
Spring CWB * ΔT	0.05	0.022	2.34	**0.019**

Drought in spring had a positive effect, while drought in summer had no effect. The model included seed production (scaled between 0 and 1 at the site level) as a response, while masting cue (ΔT, i.e., the difference between summer temperatures two and one year before flowering), spring (April–May) and summer (June–September) climatic water balance (CWB, negative values indicate water deficit), summer CWB in year T–1, and previous year seed production (Seeds T–1), were fixed effects. The model included site ID as a random intercept and was fitted with a Tweedie error distribution and logit link function. Bold indicates statistical significance (P<0.05).

**Fig. 2.**
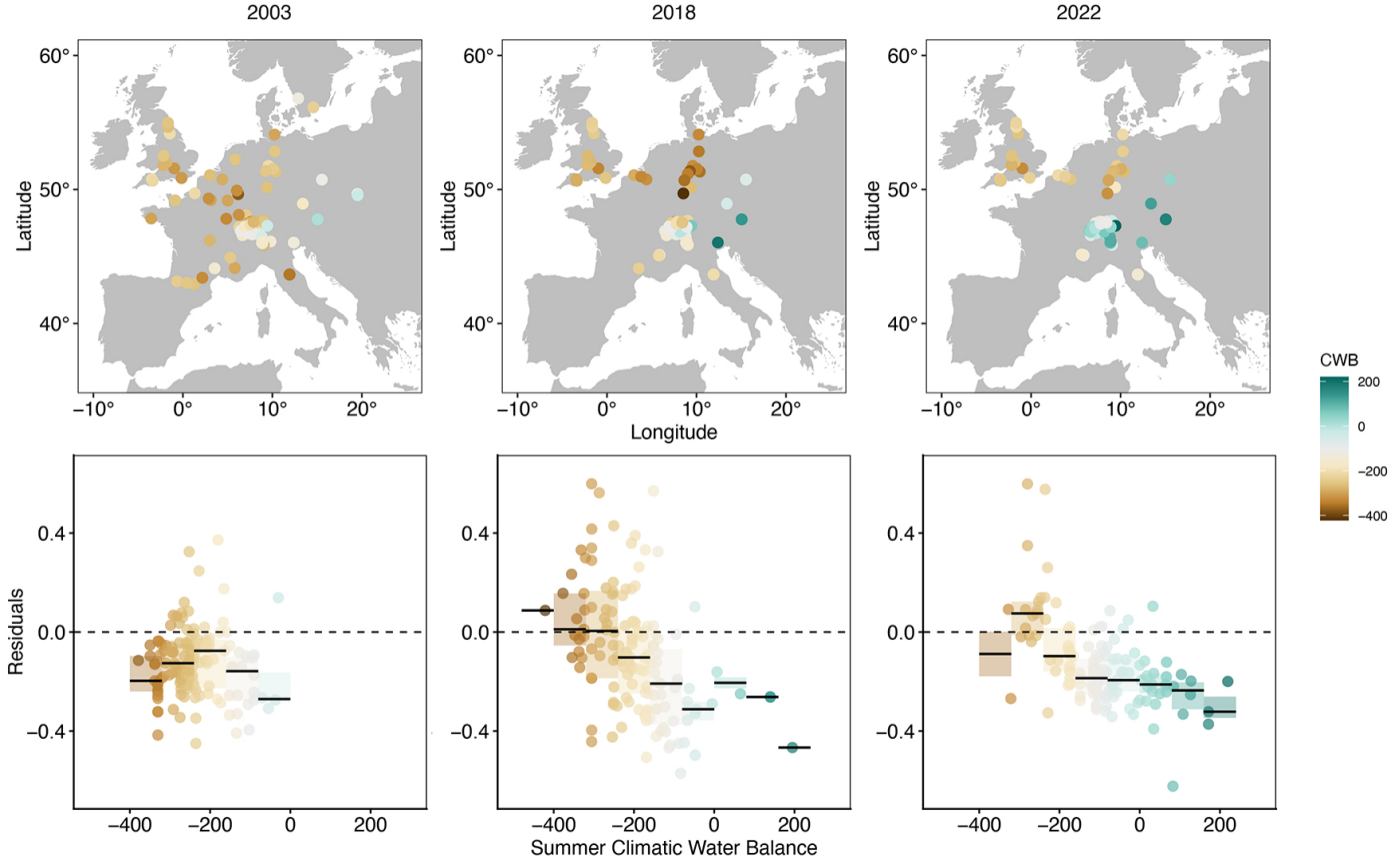
Seed production response to climatic water deficit during the severe droughts in 2003, 2018, and 2022. Points in *Top* panels show the location of sites available in MASTREE+ and Swiss Intercantonal Forest Observation databases (N=151 for 2003, N=143 for 2018, and N=127 for 2022). *Bottom* panels show the relationships between the residuals from a GLMM predicting seed production as a function of masting cues and spring drought, but keeping the effect of summer drought fixed at its median value (*Materials and Methods* and Table 1), versus summer climatic water balance of each site in a given year (CWB). Negative residuals show reproduction was lower than predicted based on cues and spring drought conditions. The bins highlight the median and interquartile range (25th and 75th) for given CWB values. The density plots of summer CWB values in 2003, 2018, and 2022 in the context of all other years in our data are shown in *SI Appendix*, Fig. S2.

A complementary analysis of extreme drought years in 2003, 2018, and 2022 corroborated these findings (Fig. 2). At a continental scale, these three droughts are the most severe European events of recent decades, and possibly of the last two millennia (8, 13, 55, 56). Nevertheless, their severity varied across our network sites (*SI Appendix*, Fig. S2). Sites experiencing the most severe drought conditions in these years did not have lower than expected seed production, based on the ΔT weather cue (Fig. 2). Indeed, in 2018 and 2022, it was the wettest sites that had lower than expected seed production, rather than the driest, where seed production was close to that predicted (negative residuals at wet sites, Fig. 2). In other words, sites that experienced the most severe conditions in these exceptional European summer droughts did not have lower-than-predicted seed production.

## Discussion

Our findings demonstrate that once European beech trees are committed to reproduction, following favorable masting cues, drought does not impair the quantity of seeds produced, even under extreme conditions. Across 221 time series, we found no evidence that drought during fruit maturation reduced seed output in the year of drought or the following year. Instead, seed production was maintained or slightly increased, including in years of severe summer drought such as 2003, 2018, and 2022. These patterns indicate that, at the stand level, reproductive output is maintained even at drought intensities that simultaneously suppress growth and elevate mortality within the same forests (7, 13, 57, 58), including at our study sites.

Our results suggest that reproduction in beech is resilient to drought, resulting from high resistance in the year of flowering and seed maturation (neutral or even positive effects of drought on seed production) and an absence of lagged suppression of reproduction the following year. The positive association between dry spring conditions and seed production is likely explained by enhanced pollen dispersal and improved pollination efficiency under low humidity, as previously observed in oak populations, where airborne pollen concentrations increased during dry conditions (45, 49). This effect is strongest when the weather cues of masting are weaker. We interpret this effect as representing greater benefits of weather-enhanced pollen dispersal in years when conspecific flowering effort is lower, and pollen limitation is otherwise greater. In beech, airborne pollen abundance has been shown to correlate with seed production (59, 60), supporting the interpretation that dry springs may facilitate higher reproductive success via improved pollen transfer.

We also found no effect of summer drought on seed production. This result suggests that, once reproduction has been initiated by prior-years temperature cues, trees continue to allocate resources to seeds even under water stress. The mechanisms governing carbon allocation among competing sinks under drought remain unresolved (22, 23, 61), and our findings indicate that developing fruits are not downregulated relative to other functions during short-term drought. Indeed, several lines of evidence suggest how beech could maintain carbon supply to developing fruits, even under severe drought. Drought limits photosynthesis via stomatal closure, which serves to avoid hydraulic failure, but does not necessarily translate immediately into carbon limitation (25, 35, 50). A delayed onset of carbon limitation may especially be the case in anisohydric species such as beech, as stomata tend to remain open for longer under drought, increasing hydraulic risk, but allowing continued gas exchange under stress (25, 35). Even when stomata are closed under severe drought to prevent runaway cavitation (62), trees possess substantial nonstructural carbon reserves (63–65), and there is little evidence that single-year droughts cause acute carbon limitation (22, 25, 35, 50, 66). Growth reduction under short-term drought is thought to result from low water potential and turgor-driven inhibition of cell expansion, alongside hormonal signaling that suppresses cambial activity, not from a lack of available carbon (50, 56, 67). In fact, increased concentration of secondary metabolites (defenses) may follow drought, likely due to decreased growth sink activity (22). Under such conditions, trees may draw on stored reserves to sustain seed maturation (51), especially if doing so offers a selective advantage. Continued investment in seeds may thus reflect both the low immediate cost of reproduction under short-term drought and the potential long-term benefit of successful recruitment in drought-disturbed environments (68, 69).

Similar responses have been documented in other species. In *Q. ilex*, *P. ponderosa*, and *P. abies*, drought has been shown to intensify trade-offs between growth and reproduction, with reproductive effort maintained despite reductions in growth (26, 40, 41), but see ref. 44 who found no trade-off, possibly because stem and fruit growth are temporally offset in their study species. While negative effects of drought on seed production have been reported in several systems (70, 71), including *Pinus sylvestris* (37), and Mediterranean oaks (27, 72), positive or neutral responses have also emerged. For example, genus-level analysis by XX (29) found that seed production in fir and pine species in the Sierra Nevada was not negatively affected by drought (*Pinus*), and in some cases even increased (*Abies*). Reduced rainfall was linked to enhanced reproductive output in *Sorbus aucuparia* (28, 73), and seed production in tropical communities also showed resilience to drought (74). These findings point to a broader pattern where reproduction may be buffered against short-term drought stress. Future studies combining drought manipulations with carbon budget tracking and reproductive monitoring are needed to clarify the mechanisms (44, 72). This will include understanding how the response of seed production to drought varies among species, and how this fits within broader physiological and ecological drought strategies (23, 75). Whether reproductive investment remains stable under multiyear droughts also remains unclear, including the potential for chronic drought or more frequent or recurrent climatic extremes to gradually erode internal carbon reserves (50, 66).

Although drought does not disrupt seed production once reproductive commitment has occurred, this does not imply that beech reproduction is broadly resilient to climate change. Other pathways threaten reproduction, particularly the increasing frequency of masting cues under warming (76). Warmer summers induce flowering more often, but frequent reproduction can outpace resource accumulation (77, 78), reducing reproductive efficiency and growth (78, 79). In the United Kingdom, the resulting breakdown of masting has lowered flowering synchrony, pollination success, and increased seed predation, cutting viable seed output by over 50% despite mean reproductive effort slightly increasing (80). Similar declines in variability are emerging elsewhere across the range (81). Thus, the absence of drought effects does not indicate resilience of seed production to climate change overall.

Moreover, reproductive resilience at the seed production stage does not guarantee successful regeneration (18, 82). Drought during maturation may reduce seed viability, such that seedling establishment, and survival, are sensitive to moisture availability and are likely to be strongly affected by climate extremes (83–86). Extreme droughts can eliminate seedling banks, sharply reducing future recruitment potential (13). Evidence from other forest systems shows that seed availability alone is insufficient to ensure regeneration: When soil moisture falls below critical thresholds, recruitment may fail even if seeds are available (85). These effects are species-specific. Experimental heating crossed with watering in North American forests demonstrated strong contrasts: Seedling survival of *Picea engelmannii* declined drastically under warming, whereas *Pinus flexilis* showed little response, resulting in simulated rapid range contraction of the former but not the latter (87, 88). Water additions ameliorated these effects, highlighting the pivotal role of drought. How these dynamics operate in beech and European forests more broadly remains poorly understood, and further work is needed to assess the sensitivity of recruitment to drought (25) and the interaction between seed supply and postdispersal climatic conditions.

Our analysis isolates the response of seed production to summer drought during the seed maturation phase and demonstrates that once flowering has been triggered, European beech maintains reproductive output even under extreme water deficits. While our data prevent analysis of spatial variation in fecundity, differences across moisture regimes may exist and could reflect local adaptation of reproductive strategies to prevailing hydrological conditions (89, 90). Identifying such patterns could help clarify whether reproductive resilience is uniform across the species’ range or shaped by long-term environmental constraints, with implications for predicting demographic responses under future climates (90, 91). Likewise, while our study focuses on short-term drought events, understanding how beech reproduction responds to chronic or multiyear water stress remains an important next step. Evidence from other systems indicates that prolonged drought can depress reproductive output over time (92). With climate projections indicating increasing frequency and severity of dry periods, assessing reproductive responses to long-term stress should be a research priority (93). Notably, while our dataset does not include the southernmost range margin of the species where moisture limitation is most severe, it includes well-characterized episodes of extreme drought, including the record European droughts of 2003, 2018, and 2022, with sites within our network experiencing summer climatic water balance deficits below −300 mm, and where abundant evidence of strong effects on tree physiology and forest functioning have previously been documented (Fig. 2) (7, 13, 55, 58), ensuring that our results reflect tree responses under genuine drought conditions. The resilience of reproduction potentially comes at the cost of increased drought-related mortality, if sustained reproduction speeds up carbon depletion (78, 94). Further work is needed to assess responses under chronic drought, variation across moisture regimes, and the demographic consequences of sustained reproduction under stress.

## Materials and Methods

### Data.

#### Seed production.

**Fig. 3.**
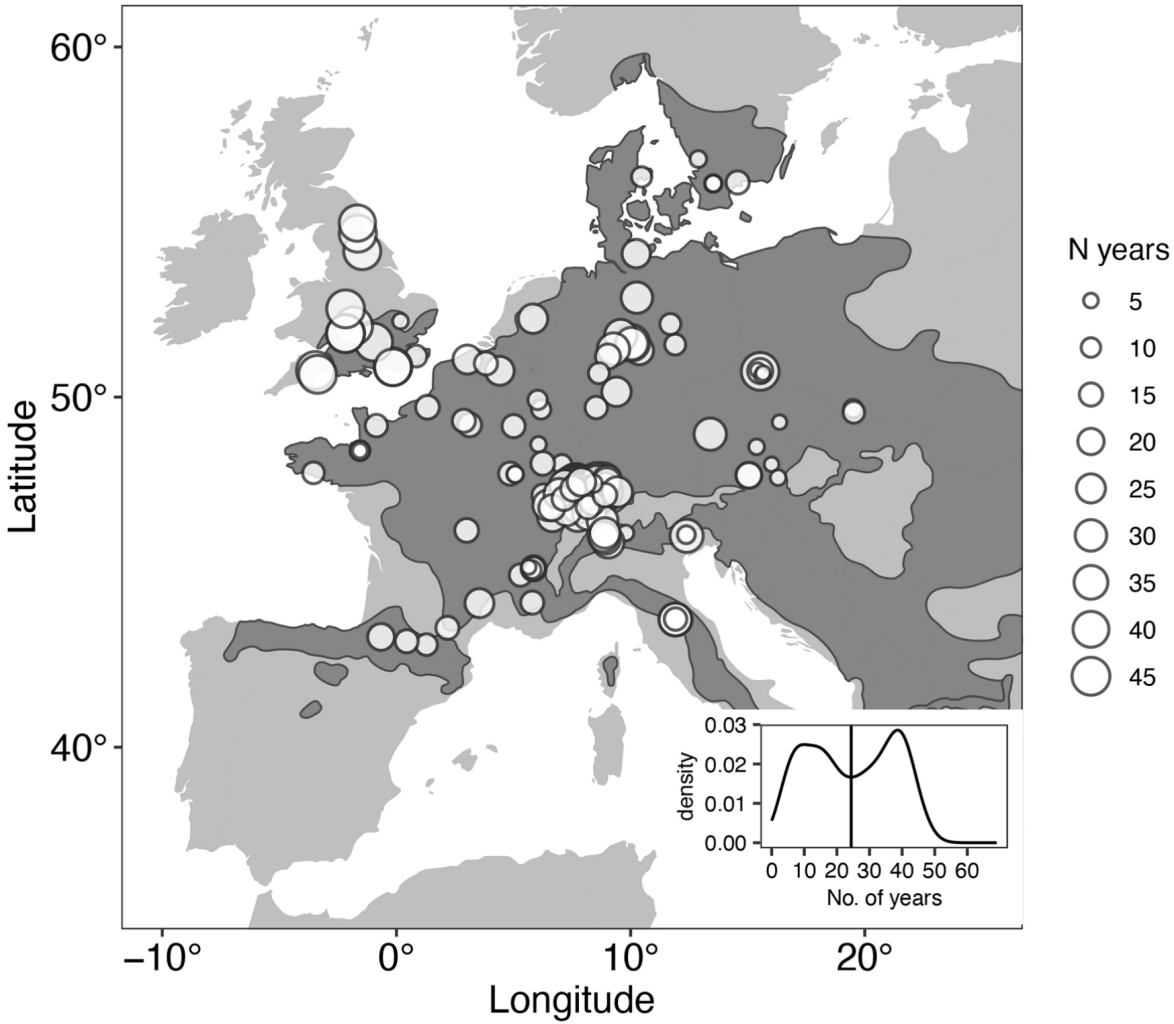
Map of study sites. Locations of the 221 time series of annual seed production of European beech (*Fagus sylvatica*) used in this study (N=5,362, average *N* per site = 24; see the *Inset* plot for how sample size was distributed across sites). Size of points is scaled to the number of observations at the focal site. The shaded area highlights the species range, based on EUFORGEN (97).

Annual observations of European beech seed production were obtained from MASTREE+, an open-access database of annual records of population-level reproductive effort (81, 95). We selected European beech because its masting cues are well studied and consistently linked to the summer solstice (48), providing a clear temporal reference point that facilitates alignment of reproductive cues with climatic drivers across populations throughout the species’ range. Only continuous time series longer than 5 y and beginning in 1952 or later were included, ensuring both sufficient length and overlap with climatic records. Pollen-based, ordinal, and regional-scale records were excluded. For several sites with ongoing monitoring, we supplemented MASTREE+ records with additional observations, extending coverage beyond 2019. Additionally, we added 105 sites from the Swiss Intercantonal Forest Observation network, where annual fruit production is estimated using fruit counts and fruit scars (58). Importantly, our seed, fruit, and fruit scar datasets do not record viable seed production, and are a measure of fruiting effort. The dataset cannot resolve seed viability or seed quality, including the presence of empty seeds (parthenocarpy). Thus, in total, our dataset included 221 series, with an average length of 24 y and a total of 5,362 observations (Fig. 3) (96). The sample for the three exceptional drought years (2003, 2018, 2022) was 151, 143, and 127 observations, respectively.

#### Climate.

We extracted daily climate data for each study site from the corresponding 0.1^°^ grid cell of the E-OBS dataset (98). From these records, we derived daily temperature and precipitation. We quantified drought as the climatic water balance (CWB), calculated as monthly precipitation sum minus potential evapotranspiration, summed for April–May (spring) and June–September (summer). Potential evapotranspiration was estimated using the Thornthwaite method. CWB reflects the balance between water supply and demand but does not simulate local soil moisture availability. Additionally, in mature trees capable of deep rooting, access to deeper water sources can temporarily decouple tree water-status from CWB deficits. We also quantified drought using vapor pressure deficit (VPD), a measure of atmospheric dryness, calculated from daily mean temperature and relative humidity following ref. 99, and aggregated to monthly means for the same seasonal windows.

### Analysis.

To test the effects of drought on annual seed production, we fitted a generalized linear mixed model (GLMM) with a Tweedie distribution and logit link, including site ID as a random intercept. The Tweedie distribution was chosen because it accommodates zero inflation and overdispersion, both of which are common features of seed production data. The response variable was annual site-level observations of population seed production. To account for differences in monitoring methods among sites in MASTREE+, seed production values were standardized within each site to range between 0 and 1 (48, 100).

Fixed effects included spring climatic water balance (CWB; April–May), summer CWB (June–September), and the difference in mean maximum summer temperatures between the year one and two years prior to seedfall (ΔT). The latter captures the established cueing system of European beech reproduction: low June–July temperatures two years before seedfall (T2) followed by high temperatures one year before seedfall (T1) (77). Because these cues are anchored to the summer solstice and are consistent across the species’ range (48), ΔT provides a parsimonious representation of masting drivers, combining T1 and T2 into a single parameter (101). We also included the lagged effect of summer CWB (CWBT_*T*−1_) to test for lagged effects of summer drought on seed production. We included seed production in the previous year (T–1), following standard practice in masting studies, to account for short-term legacy effects associated with resource depletion (102). The full model included two interactions, between ΔT and spring CWB, and between ΔT and summer CWB. We removed nonsignificant interactions from the final model.

To ensure comparability across sites and to separate within-site temporal variation from among-site spatial differences, all predictors were standardized by subtracting their site-level mean and dividing by the site-level SD (z transformation) (56). Working with anomalies provides the advantage of accounting for local adaptation and acclimation, as site-specific means capture baseline climatic conditions while highlighting deviations relevant to physiological responses. However, because CWB was standardized within sites, years that are relatively dry compared to local averages may be classified as negative anomalies even if absolute CWB remains positive (i.e., precipitation still exceeds potential evapotranspiration). Thus, anomaly-based drought metrics can reflect relative dryness rather than absolute water deficit (7). We additionally tested an alternative approach, in which CWB was entered as observed, and supplemented with site-level mean CWB to avoid mixing within- and among-site level variation (103). In these models, we also added an interaction term between site-level mean and annual variation in CWB, to test whether responses to drought vary with local moisture norms. That interaction was not significant in the case of summer CWB (*SI Appendix*, Table S1); thus, the response to summer drought was consistent across space. In the case of spring CWB, the driest sites were characterized by the strongest positive responses to dry springs (*SI Appendix*, Table S1). Nonetheless, we report the simpler z-transformed models in the main text. Models using vapor pressure deficit (VPD) instead of CWB produced qualitatively similar outcomes (*SI Appendix*, Table S1 and Fig. S1). Model validation by graphical exploratory inspection of residual patterns indicated normality and homogeneity (*SI Appendix*, Fig. S3). We also estimated Variance Inflation Factor values for our models, which indicated lack of collinearity issues. Residual spatial autocorrelation was checked and was absent.

#### Extreme drought years.

In a complementary analysis, we examined the effects of extreme summer droughts (2003, 2018, and 2022). The growth, vitality, and mortality response of European forests to these events has been the subject of intense prior investigation (7, 11–13, 15, 55, 57, 58). The spatial extent and severity of summer CWB varied across Europe for each event, and sites in our network experienced a range of conditions, including sites with strong summer CWB deficits (*SI Appendix*, Fig. S2). For each year, we compared observed seed production to model predictions while holding summer CWB at each site’s median conditions. We examined how model residuals varied with local summer CWB to test whether seed production was overestimated in sites experiencing the most severe drought in these years, in comparison to sites in our network that did not experience drought in those years.

We conducted our analysis in R version 4.2.3 (104), using the *glmmTMB* package to fit the model (105), the *SPEI* package to calculate climatic water balance (106), and the *plantecophys* package to calculate vapor pressure deficit (99).

## Supplementary Material

Appendix 01 (PDF)

## Data Availability

Data and R scripts have been deposited in OSF (https://doi.org/10.17605/OSF.IO/KFV7Y) (96).
