# Supplementary material for: European beech reproduction is not reduced by drought, including the 2003, 2018, and 2022 extremes
Source: Proc Natl Acad Sci U S A. 2026 Jun 29;123(27):e2607167123. doi: 10.1073/pnas.2607167123 (PMC13342953; doi:10.1073/pnas.2607167123)
Supplement: Supplementary file 1 — Appendix 01 (PDF) [file pnas.2607167123.sapp.pdf]

Supplementary Information: European beech reproduction is not reduced by drought, including the 2003, 2018, and 2022 extremes

Jakub Szymkowiak[1,2], Michał Bogdziewicz*[1], Dave Kelly[3], Jessie Foest[1], Sabine Braun[4], Burkhard Beudert[5], Francesco Chianucci[6], Andrea Cutini[6], Rachel Gaulton[7], Georg Gratzer[8], Angelika Kölbl[5], Georges Kunstler[9], Jonathan G. A. Lageard[10], Henning Meesenburg[11], Francesco Mezzavilla[12], Martina Mund[13], Anita Nussbaumer[14], Mario B. Pesendorfer[8], Wolfgang Schmidt[15], Anne Thimonier[14], Peter A. Thomas[16], Stanislav Vacek[17], Zdeněk Vacek[17], Arne Verstraeten[18], Markus Wagner[11], Andrew Hacket-Pain*[19]

[1]Forest Biology Center, Institute of Environmental Biology, Faculty of Biology, Adam Mickiewicz University, Uniwersytetu Poznańskiego 6, 61-614 Poznań, Poland.

[2]Population Ecology Research Unit, Institute of Environmental Biology, Faculty of Biology, Adam Mickiewicz University, Uniwersytetu Poznańskiego 6, 61-614 Poznań, Poland.

[3]School of Biological Sciences, University of Canterbury, Christchurch, New Zealand.

[4]Institute for Applied Plant Biology, Witterswil, Switzerland.

[5]Department of Conservation and Research, Bavarian Forest National Park, Grafenau, Germany.

[6]CREA – Research Centre for Forestry and Wood, Arezzo, Italy.

[7]Fera Science Ltd, York Biotech Campus, Sand Hutton, York, YO41 1LZ, United Kingdom.

[8]BOKU University, Institute of Forest Ecology, Department of Ecosystem Management, Climate and Biodiversity, Peter-Jordan-Strasse 82, A-1190 Vienna, Austria

[9]Université Grenoble Alpes, INRAE, LESSEM, Saint-Martin-d'Hères, France.

[10]Department of Natural Sciences, Manchester Metropolitan University, Manchester M1 5GD, United Kingdom.

[11]Department of Environmental Control, Northwest German Forest Research Institute, Göttingen, Germany.

[12]Via Malviste 4 – 31057 Silea (TV), Italy.

[13]Forestry Research and Competence Centre Gotha, Gotha, Germany.

[14]Swiss Federal Institute for Forest, Snow and Landscape Research WSL, Birmensdorf, Switzerland.

[15]Department of Silviculture and Forest Ecology of the Temperate Zones, University of Göttingen, Göttingen, Germany.

[16]School of Life Sciences, Keele University, Staffordshire ST5 5BG, United Kingdom.

[17]Faculty of Forestry and Wood Sciences, Czech University of Life Sciences Prague, Prague, Czech Republic.

[18]Research Institute for Nature and Forest (INBO), Geraardsbergen, Belgium.

[19]Department of Geography and Planning, School of Environmental Sciences, University of Liverpool, Liverpool, United Kingdom.

*corresponding authors: michalbogdziewicz@gmail.com; Andrew.Hacket-Pain@liverpool.ac.uk

Table S1: Results of the Generalised Linear Mixed model testing for the effects of seasonal drought, quantified using Vapour Pressure Deficit (VPD), on European beech reproduction. Drought in spring had a positive effect, while drought in summer had no effect. The model included seed production (scaled between 0 and 1 at the site level) as a response, while masting cue (ΔT, i.e., the difference between summer temperatures two and one year before flowering), spring (April-May) and summer (June-September) Vapour Pressure Deficit (VPD), and previous year summer VPD and seed production (seeds T-1), were fixed effects. The model included siteID as a random intercept and was fitted with a Tweedie error distribution and logit link function. The interaction between ΔT and summer VPD was not significant (z = 0.72, p = 0.474), therefore dropped from the model.

Term	Estimate	SE	z	p
Intercept	-1.38	0.0399	-34.58	**<0.001**
ΔT	0.71	0.0241	29.22	**<0.001**
Spring VPD	0.27	0.0199	13.47	**<0.001**
Summer VPD	-0.13	0.0200	-6.32	**<0.001**
Summer VPD T-1	0.23	0.0256	9.04	**<0.001**
Seeds T-1	-0.58	0.0207	-27.80	**<0.001**
Spring VPD * ΔT	-0.13	0.0253	-5.32	**<0.001**

Table S2: Results of the Generalised Linear Mixed model testing for the effects of site-level mean climatic water balance (CWB) in spring and summer on the effects of drought-reproduction relationship in European beech. The model included seed production (scaled between 0 and 1 at the site level) as a response, while site-level mean CWB in spring (April-May) in interaction with annual spring CWB, and mean summer (June-September) CWB in interaction with annual summer CWB, were fixed effects. The model included also masting cue (ΔT, i.e., the difference between summer temperatures two and one year before flowering) and previous year seed production (seeds T-1) as fixed effects, while siteID was random intercept. The model was fitted with a Tweedie error distribution and logit link function. The interaction between site-level mean summer CWB and summer CWB was not significant (z = -0.78, p = 0.436), therefore dropped from the model.

Term	Estimate	SE	z	p
Intercept	-1.017	0.08	-13.02	**<0.001**
Mean Spring CWB	-0.004	0.001	-3.50	**<0.001**
Spring CWB	-0.003	0.0004	-7.34	**<0.001**
Mean Summer CWB	0.002	0.0006	3.87	0.160
Summer CWB	-0.0003	0.0002	-1.40	**<0.001**
ΔT	0.530	0.01	46.54	**<0.001**
Seeds T-1	-0.001	0.00006	-10.73	**<0.001**
Mean Spring CWB * Spring CWB	0.00001	0.000004	2.67	**0.008**

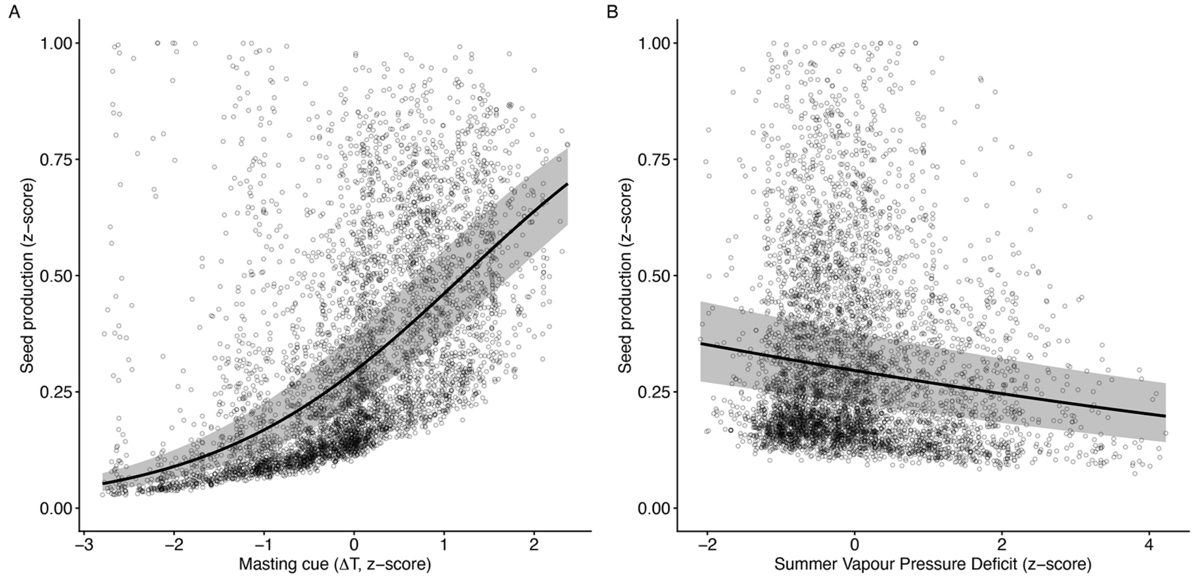

Figure S1: European beech reproduction is not suppressed by seasonal drought. Estimated relationships between A) masting cue (T, i.e., difference between summer temperatures two and one year before flowering, see Methods) and B) summer (July-September) Vapour Pressure Deficit (VPD) on population-level seed production in European beech. Points show partial residuals of a model including the T masting cue, spring VPD, summer VPD and prior year summer VPD and seed production (see Methods).

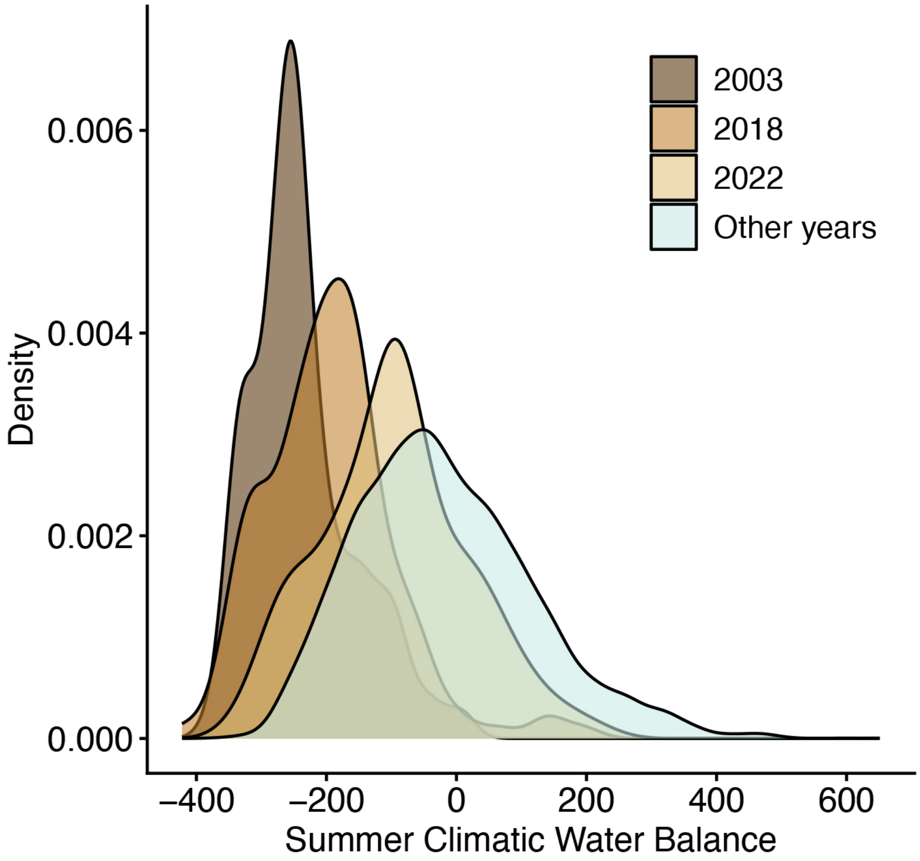

Figure S2: **Drought severity in the 2003, 2018, and 2022**. Density plots showing variation in drought severity as measured by Climatic Water Balance (CWB) in 2003 (n = 151), 2018 (n = 143), and 2022 (n = 127) in the context of all other years in the data (1967 to 2023).

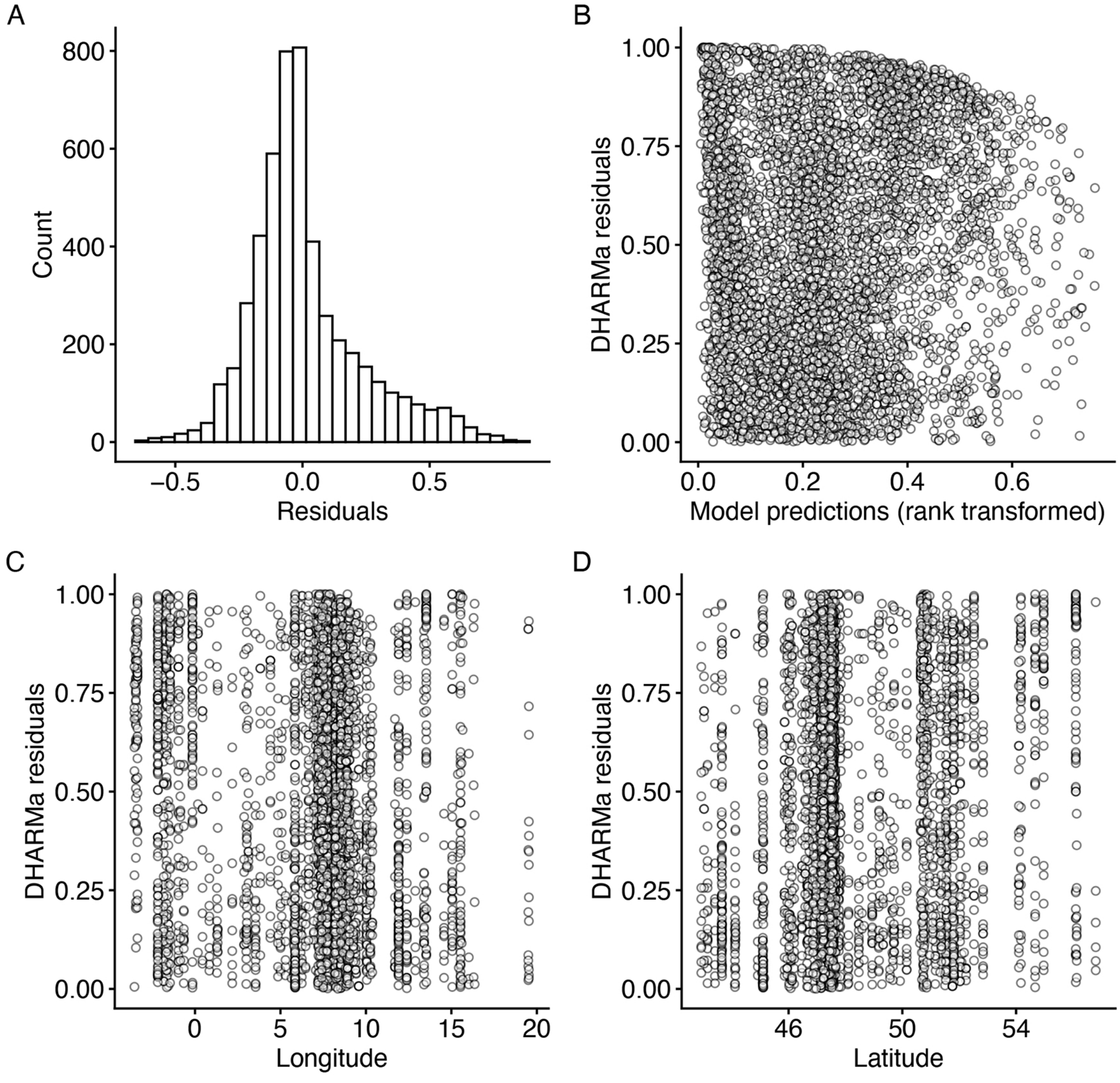

Figure S3: Model diagnostics. Model validation by graphical exploratory inspection of residual patterns indicated normality (A), variance homogeneity (B), and lack of spatial patterns in residuals (C, D).
